# Molecular Pathways Engaged by Immunomodulatory Agents in Monoclonal Gammopathy-Associated Pure Red Cell Aplasia Rescue

**DOI:** 10.3389/fonc.2020.01490

**Published:** 2020-08-27

**Authors:** Rakesh Verma

**Affiliations:** Yale Cancer Center, Yale University, New Haven, CT, United States

**Keywords:** MGUS, multiple myeloma, IMiDs, immunomodulation, ikaros (IKZF1), CRBN

## Introduction

Anemia remains a challenge for most cancer patients treated with therapies that include chemotherapy to current immunotherapy or chemo-immunotherapy treatments. Multiple myeloma (MM) is a plasma cell neoplasm marked by the clonal proliferation of malignant plasma cells. Preceding stage in MM includes asymptomatic stages including monoclonal gammopathy of undetermined significance or MGUS before the disease progression to clinical MM. One of the clinical features of MM at diagnosis includes anemia, presenting in a majority of MM patients. Pathological changes in bone marrow microenvironment in monoclonal gammopathy/MGUS and MM contribute to the pathological imbalance in erythroid cell production, leading to lowered downstream mature erythroid cells. Molecular events and pathways that contribute to this pathological remodeling of erythroid pathway-elements remain unclear and need further investigations.

## Anemia and Myeloma Progression

Anemia is among the key clinical hallmarks of myeloma diagnosis and also requires clinical management in patients with uncontrolled active progressive disease (1). Presentation of clinical myeloma involves preceding asymptomatic stages including monoclonal gammopathy of undetermined significance. Data on monoclonal gammopathy or MGUS associated pure red cell aplasia (PRCA) has remained obscure and requires more attention beyond a few recent reports (2). Pure red cell aplasia is marked by absence of precursor erythroid cells or erythroblasts, eventually leading to erythroid hypoplasia in the bone marrow of these patients. Novel targets including **R**egulator of **H**emoglobinization and **E**rythroid cell e**X**pansion or RHEX in the erythropoietin (EPO)/EPO receptor (EPOR) pathway that play key roles in erythroid cell maturation and development exclusively in humans were recently reported (3). Molecular mechanisms underlying the development of PRCA remain largely unidentified with description of immune-mediated either humoral or cellular ablation of erythroid precursors in the bone marrow. This translates into inefficient erythropoiesis leading to requirement of frequent blood transfusions to rescue anemia in these MGUS and MM patients.

**Figure 1:**
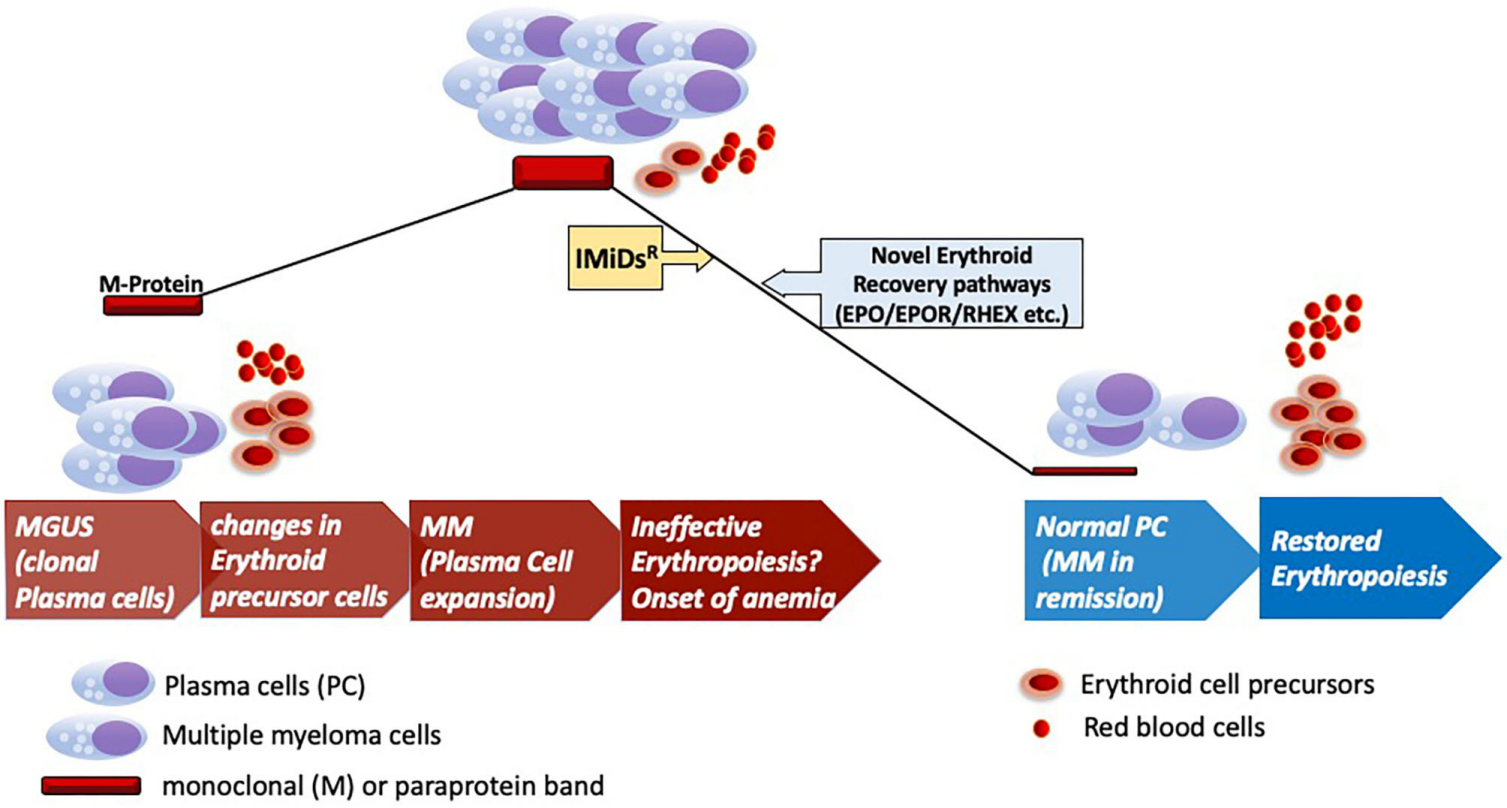
Erythroid recovery in MGUS and MM. Development of clinical myeloma (MM) from preceding MGUS stage of the disease marking onset of pathological changes in the bone marrow for the onset of anemia. Anemia marks of the key features of clinical myeloma as shown with infiltration of clonally expanded malignant plasma cells. Therapeutic intervention with IMiDs^R^ leading to disease remission and recovery of the erythroid elements in the bone marrow with accompanied changes in the microenvironment. Anemic recovery might potentially engage pathways that are dependent on factors implicated in erythroid maturation (RHEX) and EPO/EPOR pathways. *IMiDs*^*R*^*, Immunomodulatory drugs*; *EPO, Erythropoietin; EPOR, Erythropoietin Receptor*; *RHEX*, ***R****egulator of* ***H****emoglobinization and* ***E****rythroid cell e****X****pansion*.

Immunomodulatory (IMiD) agents like pomalidomide have recently been reported to induce increased *in vivo* γ-globin levels in erythrocytes of multiple myeloma patients (4). These studies have revealed the molecular mechanisms by which IMiDs like pomalidomide and lenalidomide reactivate fetal hemoglobin in myeloma patients. These pathways further help translate this activation of fetal hemoglobin into recovery from anemia clinically, while still maintaining the anti-myeloma effects of this class of IMiD^R^ drugs. Although the molecular mechanisms underlying the anti-myeloma activity of IMiDs^R^ have been reported to involve the Cullin 4A (CUL4A)- Cereblon (CRBN) E3 ligase complex mediated proteasomal degradation of downstream targets (5). These CRBN targets include transcription factors like IKAROS (IKZF1), while additional molecular pathways underlying recovery from anemia in myeloma remain IKAROS or IKZF1 independent (4–6). Specifically, transcriptional modules affected by pomalidomide or lenalidomide has been reported to involve γ-globin repression included BCL11A, SOX6, IKZF1, KLF1, and LSD1. But IKAROS (IKZF1) was not identified as the key effector of this program, as IKZF1 ablation was not sufficient to phenocopy pomalidomide treatment (4). These findings point to the existence of additional pathways that may include EPO/EPOR dependent mechanisms driving the onset and recovery from anemia in MGUS and MM patients (Figure 1).

It is to be noted that monoclonal gammopathy-associated PRCA might be a paraprotein-related phenomenon. It can be further speculated that there could be a functional relationship between altered plasma cell and erythroid precursor, with a possible causal relationship between M-protein response and hematological response including PRCA reversal in the PRCA bone marrow after treatment with pomalidomide or IMiDs (2). A number of intriguing questions regarding the underlying molecular and pathogenetic mechanisms still remain unanswered. Modulation of tumor microenvironment is aptly speculated via possible roles of IgG monoclonal protein for erythroid inhibition. But based on the roles of IKZF1 and CRBN for eliciting anti-myeloma effects for any possible hematological response accompanying reduction in the disease burden (M-protein), it remains unclear if recovery of erythroid progenitors is CRBN or IKZF1 independent. Additionally, possible changes in the γ-globin levels can be further speculated in patients with hematological recovery after anti-myeloma therapies (IMiDs^R^ based) as reported by Dulmovits et al. (4).

## Discussion

Detailed molecular mechanisms describing the additional targets of IMiD action remain an active area of research (7) and these additional pathways may contribute to the recovery of erythroid elements in the bone marrow of MM patients. This is further validated by the data on absence of expected JAK2 V617F and CALR Type 1/2 type mutations in the monoclonal gammopathy-associated PRCA cohort reported by Korde et al. (2) eliminating the dysfunctional EPOR mediated JAK2-STAT5 pathway circuitry.

Monoclonal gammopathy-associated PRCA illustrates an open ended clinical challenge to describe the underlying possible pathogenetic mechanisms by future mechanistic studies (Figure 1). Anti-myeloma drugs like IMiDs^R^ drive the erythroid recovery in this pathology but independent of currently known molecular pathways that exclusively drive the action of these drugs on myeloma cells and potentially some immune cell subsets. Absence of RTK mutations further makes a strong case for an alternative mechanistic active in the progenitor cells under the action of novel agents like pomalidomide or lenalidomide for erythroid recovery in MM. Recent discovery of novel regulators like RHEX implicated in the human erythroid cell maturation combined with the findings of Dulmovits et al. proposes further investigations of erythroid maturation pathways in MM and Monoclonal gammopathy or MGUS associated PRCA to uncover the underlying molecular mechanism. With the progress of cell therapies like the BCMA targeting CAR-T cells for late stage or relapsed/refractory MM (8), these insights into anemic recovery would benefit the ultimate advancement of advanced CAR-T based therapies for early stages of MM. Future detailed mechanistic studies can help identify alternative treatment strategies as compared to blood transfusions for these MGUS/MM patients for efficient anemic recovery.

## Author Contributions

RV prepared and wrote the manuscript.

## Conflict of Interest

The author declares that the research was conducted in the absence of any commercial or financial relationships that could be construed as a potential conflict of interest.
